# Add mass spectrometry to the pandemic toolbox

**DOI:** 10.7554/eLife.75471

**Published:** 2021-12-10

**Authors:** Bart Van Puyvelde, Maarten Dhaenens

**Affiliations:** 1 Laboratory of Pharmaceutical Biotechnology, Ghent University Ghent Belgium; 2 ProGenTomics Ghent Belgium

**Keywords:** SARS-CoV-2, Covid-19, public health, proteomics, diagnostics, mass spectrometry, Human

## Abstract

A new protocol step improves robustness and ease-of-use for mass spectrometry in the clinic, opening the door to mass deployment to monitor infectious agents.

**Related research article** Hober A, Tran-Minh KH, Foley D, McDonald T, Vissers JPC, Pattison R, Ferries S, Hermansson S, Betner I, Uhlén M, Razavi M, Yip R, Pope ME, Pearson TW, Andersson LN, Bartlett A, Calton L, Alm JJ, Engstrand L, Edfors F. 2021. Rapid and sensitive detection of SARS-CoV-2 infection using quantitative peptide enrichment LC-MS analysis. *eLife* **10**:e70843. doi: 10.7554/eLife.70843

Widespread testing has become a cornerstone of the response against the COVID-19 pandemic. So far, this has almost exclusively been done by detecting the genetic information of the SARS-CoV-2 virus (its mRNA) using tests such as RT-PCR. This approach is efficient, relatively simple and acceptably cheap. However, only relying on one type of technology can lead to supply shortages, and it makes it difficult to assess how well the method fares in terms of sensitivity, false positives and false negatives (Woolston, 2021). Additionally, while RT-PCR tests can assess whether someone is carrying the virus, they cannot reveal how many viral particles someone is releasing into the environment, how infectious a person is, or how a patient will fare. The pandemic ‘readiness toolbox’ must therefore be extended to include methods that can detect other types of biomolecules beyond mRNA, such as viral proteins and peptides (Evans et al., 2021).

Mass spectrometry, an approach that helps to assess which compounds are present in a sample, is an obvious choice. Rather than detecting a precise target (like RT-PCR assays do with mRNA), this intrinsically versatile method measures the physical properties of any and many biomolecules, including peptides derived from proteins. Mass spectrometry can therefore monitor a practically limitless number of molecules, making it a sustainable analytical technique. In fact, an instrument calibrated to quantify certain disease-related proteins in plasma, for example, can start measuring peptides derived from a new pathogen in next to no time (Grossegesse et al., 2020; Van Puyvelde et al., 2021).

However, despite the high-level sensitivity and accuracy of mass spectrometry, clinical settings often rely on other techniques to analyze proteins, as extensive expertise is believed to be needed both to handle the instrument and to interpret the data. Most of these concerns are due the additional, meaningless data from all the other proteins and molecules in the sample (‘the noise’), which complicate the detection of the peptides of interest (the actual signal). Indeed, like any other analytical technique, mass spectrometry measures the signal-to-noise ratio, but the instruments cannot automatically reduce the noise from this equation. This has made mass spectrometry a frightening prospect for clinical implementation, reducing instrumental robustness and complicating data interpretation. In response, scientists usually strive to improve the signal-to-noise ratio by increasing the signal. Now, in eLife, Fredrik Edfors and colleagues at institutions in Sweden and Canada – including Andreas Hober as first author – report having tweaked how to prepare a mass spectrometry sample to prevent background noise from emerging instead (Hober et al., 2021).

The team added a new step in the protocol, called peptide immuno-enrichment, which involves attaching antibodies to magnetic beads to ‘fish’ the target peptides directly out of the patient samples before analysis (Razavi et al., 2016; Anderson et al., 2004). This multiplies the sensitivity many-fold and reduces measurement time, interferences and instrument contamination while also increasing robustness (as also proposed in Van Puyvelde et al., 2021). Additionally, without the noise, mass spectrometry can determine the amount of a given target protein extremely accurately, something that is not possible using RT-PCR (Evans et al., 2021).

In other words, Hober et al. have set the stage for applying mass spectrometry to accurately quantify SARS-CoV-2 peptides in a variety of patient samples. With this approach, each instrument could process samples from 500 patients in a single day. In fact, with peptide immuno-enrichment already used to assess a panel of inflammation proteins in plasma in the clinic, adding an antibody bead targeting a peptide derived from a pathogen does not change the overall workflow (Anderson et al., 2020). The complete sample preparation protocol with peptide immuno-enrichment could be done for less than 40€ per sample, in less than three hours of automatable sample preparation and without requiring any mass spectroscopy expertise to interpret the data. Beyond this versatility, another important characteristic of the approach proposed by Hober et al. is that the ‘fishing’ of the target peptides theoretically means that patient samples could be pooled and analysed together. As suggested for RT-PCR (Verwilt et al., 2020), this strategy would increase throughput and save resources.

Taken together, these features provide an opportunity to develop an early warning test that targets a dozen respiratory pathogens in pools of up to 32 patient samples, with each machine being able to run 500 pooled samples – totaling up to 16,000 individuals a day. Doing this throughout the year would help to monitor how a pathogen spreads through a population over time. In fact, if several pathogens start to pass through a population simultaneously, other mass spectrometry instruments can easily be set up to detect these infectious agents in parallel in unpooled samples (up to 500 patients a day per instrument).

The question then becomes: should we now start using mass spectrometry instead of RT-PCR in the present COVID-19 pandemic? Currently, precisely assessing the amount of viral proteins may not help to decide whether a person should be quarantined. However, this information may help to decide when an individual should be released into the community as viral protein might more closely track infectivity in SARS-CoV-2 than viral mRNA, which remains detectable after a patient ceases to be infectious (Evans et al., 2021). Irrespectively, starting to measure viral protein loads in thousands of COVID-19 patients using mass spectrometry could help the technique to mature into a reliable approach to add to the pandemic readiness toolbox; in this effort, the work by Hober et al. will help revolutionize the way mass spectrometry is approached in the clinic.
